# Deriving stage at diagnosis from multiple population-based sources: colorectal and lung cancer in England

**DOI:** 10.1038/bjc.2016.177

**Published:** 2016-06-21

**Authors:** S Benitez-Majano, H Fowler, C Maringe, C Di Girolamo, B Rachet

**Affiliations:** 1 Cancer Research UK Cancer Survival Group, London School of Hygiene and Tropical Medicine, London, UK

**Keywords:** cancer, stage, TNM, routine data, survival, population-based

## Abstract

**Background:**

Stage at diagnosis is a strong predictor of cancer survival. Differences in stage distributions and stage-specific management help explain geographic differences in cancer outcomes. Stage information is thus essential to improve policies for cancer control. Despite recent progress, stage information is often incomplete. Data collection methods and definition of stage categories are rarely reported. These inconsistencies may result in assigning conflicting stage for single tumours and confound the interpretation of international comparisons and temporal trends of stage-specific cancer outcomes. We propose an algorithm that uses multiple routine, population-based data sources to obtain the most complete and reliable stage information possible.

**Methods:**

Our hierarchical approach derives a single stage category per tumour prioritising information deemed of best quality from multiple data sets and various individual components of tumour stage. It incorporates rules from the Union for International Cancer Control TNM classification of malignant tumours. The algorithm is illustrated for colorectal and lung cancer in England. We linked the cancer-specific Clinical Audit data (collected from clinical multi-disciplinary teams) to national cancer registry data. We prioritise stage variables from the Clinical Audit and added information from the registry when needed. We compared stage distribution and stage-specific net survival using two sets of definitions of summary stage with contrasting levels of assumptions for dealing with missing individual TNM components. This exercise extends a previous algorithm we developed for international comparisons of stage-specific survival.

**Results:**

Between 2008 and 2012, 163 915 primary colorectal cancer cases and 168 158 primary lung cancer cases were diagnosed in adults in England. Using the most restrictive definition of summary stage (valid information on all individual TNM components), colorectal cancer stage completeness was 56.6% (from 33.8% in 2008 to 85.2% in 2012). Lung cancer stage completeness was 76.6% (from 57.3% in 2008 to 91.4% in 2012). Stage distribution differed between strategies to define summary stage. Stage-specific survival was consistent with published reports.

**Conclusions:**

We offer a robust strategy to harmonise the derivation of stage that can be adapted for other cancers and data sources in different countries. The general approach of prioritising good-quality information, reporting sources of individual TNM variables, and reporting of assumptions for dealing with missing data is applicable to any population-based cancer research using stage. Moreover, our research highlights the need for further transparency in the way stage categories are defined and reported, acknowledging the limitations, and potential discrepancies of using readily available stage variables.

Stage at diagnosis is a key predictor of cancer survival (Richards, 2009). Differences in stage are believed to be one of the main drivers of disparities in cancer survival between and within regions (Sant *et al*, 2003). England is known to lag behind in cancer survival in comparison to other comparably wealthy countries with a universal health system (Coleman *et al*, 2011). Part of this survival differential is presumably due to a poorer stage distribution of cancer cases in England (Sant *et al*, 2003; Walters *et al*, 2013b). In the past couple of decades, many resources have been invested in improving cancer outcomes through identifying and treating cancer at an earlier stage (Richards, 2009; Department of Health, 2011).

Research examining national and international temporal and geographical patterns in cancer outcomes is usually based on population-based cancer registry data, which have historically lacked information on stage. Further granularity of information is required to understand in depth the effect of stage on cancer outcomes at the population level and to monitor and evaluate cancer policy and changes in clinical practice. Recent efforts by Public Health England (PHE) and the National Cancer Registration Service have driven an improvement in availability of stage information for cancers diagnosed in England (McPhail *et al*, 2015). The national aim is for at least 70% of cancer patients to be staged at diagnosis (Health and Social Care Information Centre, 2015).

Clinical or surgical quality assurance programmes, also called clinical audits, have been developed as instruments to ensure clinical quality standards of health-care providers (van Gijn *et al*, 2010). Clinical audits contain detailed clinical data, including information on diagnostic investigations, stage at diagnosis, and treatment for cancer (van Gijn *et al*, 2012). Besides helping clinical specialists improve their practice, clinical audits offer a rich, complementary source of clinical data for population-based cancer research.

Comparability of stage information from different sources has been a controversial issue, especially when making international or temporal comparisons, as clinical protocols, data collection methods, coding practices, and tumour classification systems may vary between geographies and time periods (Walters *et al*, 2013a). Inconsistencies may also occur between different sources of information from the same country.

We describe an algorithm to derive stage at diagnosis from different sources, based on a series of hierarchical rules applied on both the data sources and the individual stage variables from the TNM classification (Sobin *et al*, 2009). This extends the algorithm proposed and used by Walters *et al* (2013a) for the International Cancer Benchmarking Partnership module 1 study. The algorithm is illustrated for colorectal and lung cancer.

## Materials and Methods

### Data sources

The National Cancer Registry data provides information on date of birth, sex, vital status, date of death, tumour site, and morphology (Office for National Statistics, 2015).

The Cancer Analysis System (CAS) is a national database administered by the National Cancer Intelligence Network of PHE. It combines the National Cancer Registry data with data from other sources (Health and Social Care Information Centre, 2015) and holds information on main tumour features, socio-demographic characteristics, stage, and treatment dates.

**Figure 1:**
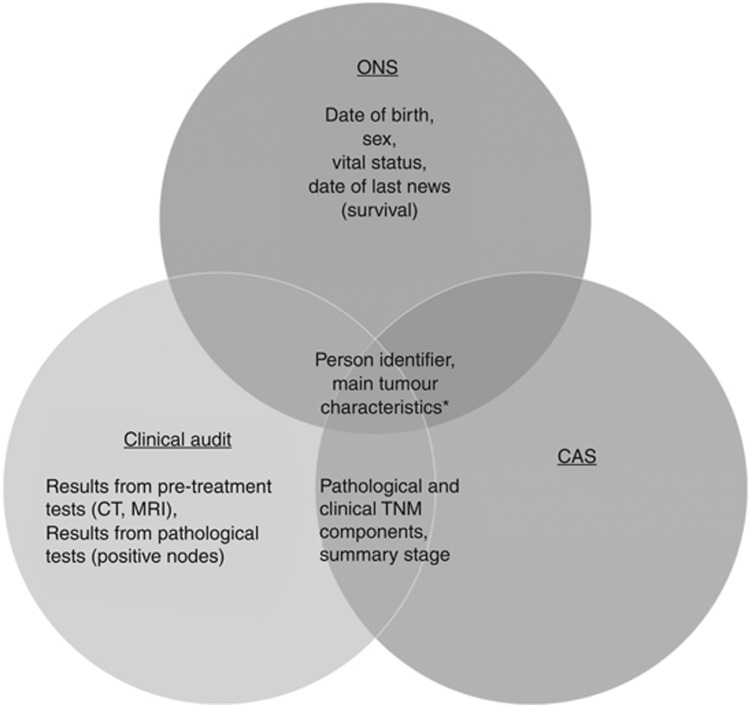
**Sources of data for deriving stage for colorectal and lung cancer, England, 2008–2012.** *Main tumour characteristics include ICD-10 topography codes, histology, behaviour, and date of diagnosis. Abbreviations: CAS=Cancer Analysis System; CT=computarised tomography scan; MRI=magnetic resonance imaging; ONS=Office for National Statistics Cancer Registration Dataset.

The National Clinical Audit Programme comprises multiple clinical audits to monitor and evaluate health-care practice on specific conditions, benchmark performance, and inform patients and the general public of current standards of care in different medical specialties (Healthcare Quality Improvement Partnership, 2015). Cancer clinical audits contain information on patient referral, diagnostic investigations, pretreatment staging, treatment, pathology evaluations, posttreatment follow-up, and outcomes. Information is collected at the hospital level and its accuracy and completeness should, in principle, be ensured by relevant clinicians before submission to the Audit (Scott *et al*, 2014). The National Bowel Cancer Audit Project (NBOCAP) was developed to collate detailed clinical bowel cancer data by the Association of Coloproctology of Great Britain in 2001 (Health and Social Care Information Centre, 2014). The Lung Cancer Audit Database (LUCADA) was developed by the Royal College of Physicians Intercollegiate Lung Cancer Group in 2002 and started collecting lung cancer data nationally in 2005 (Royal College of Physicians, 2015). Figure 1 summarises the sources of information for the stage algorithm.

### Data linkage

Individual colorectal and lung cancer records from the ONS National Cancer Registry data were linked to the CAS records of the same cancers diagnosed between 2008 and 2012. It followed a two-part strategy, linking records at the patient level using an eight-level hierarchy based on the availability of information on NHS number, date of birth, sex, and postcode and linking records at the tumour level by tumour site and diagnosis date. Of the 163 915 colorectal cancer cases (ICD-10 C18-C19) in the ONS National Cancer Registry data diagnosed in England during the study period, 158 953 (96.97%) linked to a CAS record and 121 707 (74.25%) linked to an NBOCAP record. For lung cancer (ICD-10 C33-C34), there were 168 158 tumours diagnosed in England during the study period. Of these, 167 236 (99.45%) linked to a CAS record and 131 540 (78.22%) linked to a LUCADA record.

### The staging algorithm

The algorithm is based on rules of the Union for International Cancer Control TNM classification of malignant tumours. The TNM classification was developed in the 1950s as an international standard for classifying malignant tumours by anatomical extent (Sobin *et al*, 2009). It aims to provide an unambiguous grouping of cancer cases for clinicians to make standardised and consistent decisions for adequate disease management.

The anatomical extent of disease is based on the assessment of three components: the extent of the primary tumour (T), the presence and extent of metastases to regional lymph nodes (N), and the presence of distant metastases (M). For each tumour, two classifications are defined: a pretreatment clinical classification (c), drawn from physical examination, imaging tests, endoscopy, or biopsy; and a pathological classification (p), after histopathological assessment of the primary tumour, removal and assessment of lymph nodes, and microscopic evaluation of distant metastases.

The TNM classification goes through periodic prospective and retrospective evaluations that lead to the development and publication of improved editions. The Fifth and Sixth Editions were published in 1997 and 2002, respectively (Sobin and Wittekind, 1997, 2002), followed by the current Seventh Edition, in effect since 2010 (Sobin *et al*, 2009). Possibly the biggest change between the latest editions was the elimination of the category Mx, previously used to denote that distant metastases could not be assessed (Sobin *et al*, 2009). This category is now considered inappropriate as clinical assessment of metastases may be based solely on physical examination (cM). Pathological Mx (pMx) may be misinterpreted and overused by pathologists when they have access to histological material to assess pT and pN, but not for pM, a frequent situation after surgery for resection of the primary tumour (Sobin and Compton, 2010). The deletion of this category encourages the use of M0 when metastasis cannot be proven and should facilitate the completeness of stage grouping.

### Hierarchies of data sources and stage variables

A hierarchy of data sources was established for different types of information to avoid inconsistencies, given that information could potentially come from a maximum of three data sets. The ONS National Cancer Registration data was our preferred data set for main person and basic tumour characteristics such as date of birth, vital status, deprivation quintile, topography, and morphology of the primary tumour. This decision was based on their established quality-control processes to verify this information and to be consistent with official national cancer statistics (Office for National Statistics, 2015). Clinical audits were the preferred source for detailed clinical data such as pretreatment diagnostic investigations, dates and results of medical interventions, and staging resulting from these. Staging information from CAS was used if missing or invalid from the clinical audit.

The algorithm can be divided into two parts for descriptive purposes. The first part entails deriving individual T, N, and M components from all available sources of pathological and clinical stage information. Once individual T, N, and M components have been ascertained, the second part of the algorithm applies TNM definitions for stage grouping, to obtain the overall grouped TNM stage (I, II, III, IV). Two different strategies for deriving TNM stage grouping are described depending on the acceptable level of missing information from individual T, N, and M components.

### Deriving individual T, N, and M components

We used a set of rules to treat potentially discordant information from different sources to derive overall individual T, N, and M components from different types of variables in the data sets following TNM classification rules.

The pathological TNM classification uses information from clinical TNM and complements it using additional information from pathological evaluation (Sobin *et al*, 2009). Pathological TNM should therefore be the most complete source of staging information, at least for T and N. In our data sets, there was information for pathological and clinical individual T, N, and M components from the clinical audits and CAS plus staging information from additional variables (Table 1). We gave priority to the pathological variables over clinical ones for T and N, but cM was prioritised over pM (Walters *et al*, 2013a). Although distant metastases are not generally evaluated during surgery for resection of the primary tumour (Sobin and Compton, 2010; Walters *et al*, 2013a), pathological confirmation of metastases, from a biopsy, for example, was given priority over a negative or inconclusive result from a clinical/imaging test.

Our algorithm allowed results from medical tests and diagnostic procedures to inform individual clinical T, N, and M components when missing or with a value of zero. Similarly, records of the presence of metastases in specific organs or of regional lymph node involvement were used to inform cM or pN, respectively. Information in these additional variables was used as evidence of local, regional, and/or distant extension of disease when positive but did not rule out their presence. For example, if there was evidence of distant metastases from one of these additional variables, this replaced the value of cM to cM1; however, if there was no evidence of metastasis in that variable, it did not change the value of cM to cM0, allowing the algorithm to keep looking for information in subsequent variables.

In addition to the clinical and pathological T, N, and M components, CAS reports a third type of staging information that may come from either pathological or clinical data and may use the highest value of a particular component for a given tumour or be directly flagged by the registry. This ‘integrated' stage information was used only when exhausting all other possible sources because its algorithm was not fully documented.

We used an additional step for determining the M component to account for the fact that, although the categories Mx and pM0 do not exist in the Seventh Edition of the TNM classification, their use is still common practice: If M was still missing after looking in all potential sources, and there was indirect evidence of a clinical examination, that is information on both clinical T and N, M was assumed to be M0. Once an individual overall T, N, or M component was populated at a specific step of the algorithm, there was generally no need to look further for information of that particular component in subsequent steps of lower priority.

### Deriving the grouped TNM stage

After ascertaining individual – and unique – T, N, and M values to each tumour, the second part of the algorithm followed TNM classification definitions to categorise different combinations of T, N, and M values into TNM stage groupings. This part of the algorithm starts by examining M. Generally, for most cancer sites, including colorectal and lung, a positive M value effectively represents the maximum value of TNM stage grouping, stage IV, independently of the values of N and T. Similarly, once a positive M has been excluded, and there is a positive N, the algorithm assigns a TNM stage III to the tumour, independently of the value of T. The algorithm then evaluates subsequent subcategories in a descending order (stages II and I).

To manage the missing information within N and/or M, we applied two different strategies to derive overall stage based on the algorithm for deriving stage described by Walters *et al* (2013a). The most conservative of the two approaches, the restrictive strategy, is stricter in the sense that all three components need to be present to derive the grouped stage. In contrast, the non-restrictive strategy allows for the interpretation of missing information as an absence of metastases to the lymph nodes (N) or to distant organs (M). Additionally, after exhausting all possibilities of deriving the grouped TNM stage from individual T, N, and M components, the algorithm moves on to using the pathological and clinical summary stage information. The restrictive strategy differs in that we ignore the grouped stage variables, given that we cannot verify individual T, N, and M components from these.

### The staging algorithm applied to colorectal cancer

Both CAS and NBOCAP use the Fifth Edition of the TNM classification (Sobin and Wittekind, 1997), following guidance from the Royal College of Pathologists (Health and Social Care Information Centre, 2014). The definition of node involvement changed in later editions, specifically in that evaluation of satellite mesenteric tumour deposits use a size criterion in the Fifth Edition, while the Sixth and Seventh Editions use a shape criterion to determine the presence of mesenteric lymph node involvement (Doyle and Bateman, 2012). Subdivisions of stage categories have been added, and definitions of T4a and T4b have been reversed in the Seventh Edition (Sobin *et al*, 2009). Except for the lymph node definition change, none of the changes affect definitions of overall stage grouping categories.

NBOCAP data allowed for single tumours to have several treatment records. These records may hold conflicting information on pathological T, N, and M components, presumably measured at different points in the treatment journey. Therefore, the first part of the algorithm applied to colorectal cancer was to establish a hierarchy of NBOCAP treatment records based on their closeness to diagnosis date. As a general rule, only records with treatment procedures dated within 30 days before or after the date of diagnosis were eligible to contribute with information on pathological T, N, and M components. This was to avoid assigning values of TNM associated with restaging and/or disease progression to what we define as stage at diagnosis. Of these records with procedures dated between the ±30-day window from diagnosis, the closest one to the date of diagnosis would be given priority over information contained in subsequent treatment records, assuming it contained a valid code for that variable. In cases where multiple records of one tumour had the same procedure dates, the one with lowest values of individual T, N, and M components would be given priority, following a general rule of the TNM classification (Sobin *et al*, 2009, p. 9). Information in subsequent treatment records would only be used if such information was missing in the previous one. Information on individual clinical T, N, and M components from NBOCAP was the same in all treatment records of any single tumour, as in CAS. Additional variables with information on colorectal cancer-specific staging are listed in Table 1. The full procedure to derive individual T, N, and M components of stage for colorectal cancer is detailed in Supplementary Appendix Figures S1–S3.

**Figure 2:**
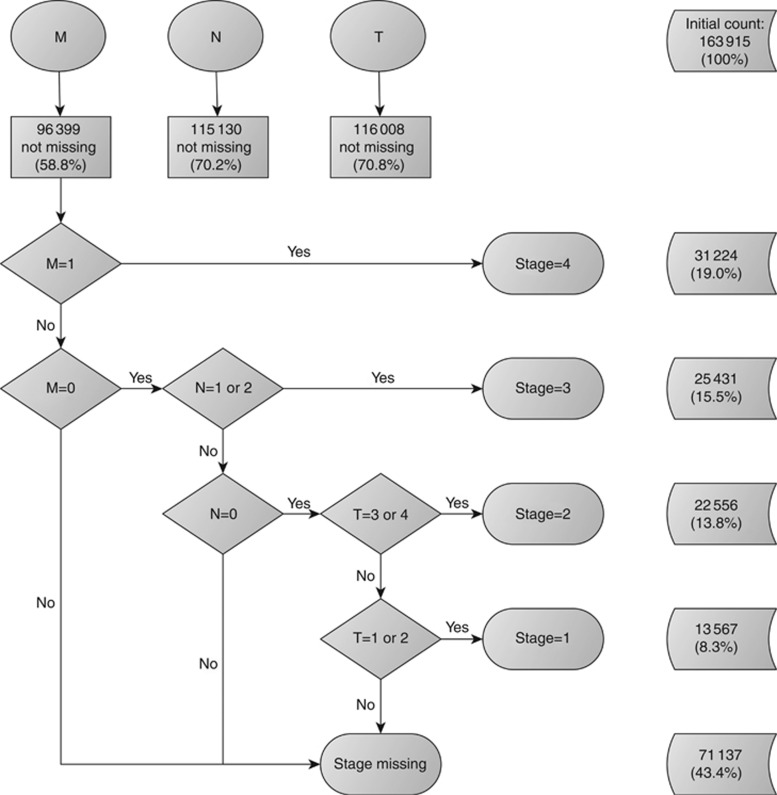
**Deriving stage for colorectal cancer using the restrictive strategy, England, 2008–2012.** Abbreviations: T=tumour; N=lymph nodes; M=distant metastases.

**Figure 3:**
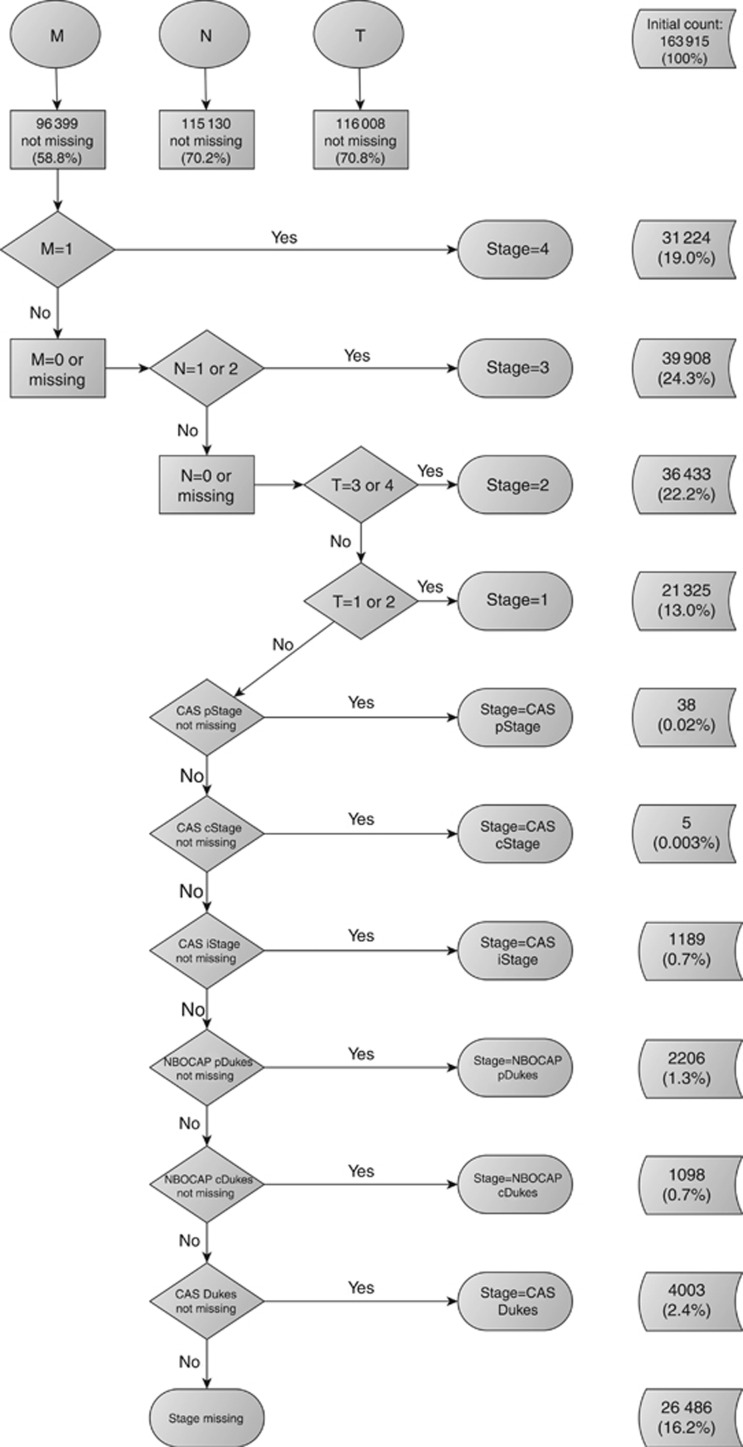
**Deriving stage for colorectal cancer using the non-restrictive strategy, England, 2008–2012.** Abbreviations: CAS=Cancer Analysis System; cDukes=clinical Dukes staging; cStage=clinical TNM stage; iStage=integrated TNM stage; M=distant metastases; N=lymph nodes; NBOCAP=National Bowel Cancer Project; pDukes=pathological Dukes staging; pStage=pathological TNM stage; T=tumour.

The second part of the algorithm for deriving overall stage grouping using the non-restrictive strategy used additional information from the colorectal cancer-specific Dukes classification. As the Dukes classification is not directly equivalent to specific combinations of individual T, N, and M components, TNM summary stage variables from CAS were given priority over Dukes staging, in the same order as individual T, N, and M variables (pathological, followed by clinical and integrated). The second part of the colorectal cancer stage algorithm is summarised in Figures 2 and 3.

### The staging algorithm applied to lung cancer

The first part of the algorithm remains as described above, except that there were no additional variables to inform individual T, N, and M components in LUCADA (See Supplementary Appendix Figures S6–S8).

The main challenge in adapting the algorithm to lung cancer was the substantive modifications between the Sixth and Seventh Editions of the TNM classification (Goldstraw *et al*, 2007): definitions of some individual components of T and M as well as of some categories of the stage grouping have changed (Mirsadraee *et al*, 2012). We derived TNM stage grouping following definitions of the Sixth and Seventh Editions of the TNM classification separately. Most of these changes do not affect the overall TNM stage grouping, therefore we chose to apply definitions of the current Seventh Edition of TNM classification for the whole study period (see Supplementary Appendix Figures S4 and S5).

### Statistical analyses

We estimated age-standardised 5-year net survival, stratified by stage, including a missing stage category, for patients diagnosed in England between 2008 and 2012 and followed up until end of 2013. Net survival represents survival with cancer as the only potential cause of death by factoring out mortality from other causes (expected mortality) (Pohar Perme *et al*, 2012). Within the relative survival setting in which causes of death are not available, the expected mortality was provided by life tables from the England general population, namely, life tables by age, sex, calendar year, and deprivation (London School of Hygiene & Tropical Medicine, 2015). Net survival was estimated with the non-parametric Pohar-Perme estimator (Pohar Perme *et al*, 2012) implemented in the Stata program *stns* (Clerc-Urmès *et al*, 2014). We used the complete approach for survival analysis, as used for national cancer survival statistics (Office for National Statistics, 2015). We used the International Cancer Survival Standard weights for age standardisation, which categorises age into five groups (15–44, 45–54, 55–64, 65–74, and 75–99 years of age) (Corazziari *et al*, 2004). We compared 5-year net survival between both versions of the staging algorithm (restrictive and non-restrictive) for each cancer.

## Results

The proportion of cases with valid information on individual T and N components was comparable between cancer sites (Table 1). Completeness of valid information on the M component varied significantly between colorectal and lung cancer (41.2% *vs* 22.8% missing M component, respectively Table 1). Of the 163 915 primary malignant colorectal tumours, 92 778 (56.6%) had valid stage using the restrictive strategy and 137 429 (83.8%) using the non-restrictive strategy. Of the 168 158 primary lung cancer cases, 128 866 (76.6%) had stage information with the restrictive strategy, *vs* 135 666 (80.7%) with the non-restrictive strategy. Completeness of derived stage improved over time for both cancer sites, as did the difference in stage completeness between the restrictive and non-restrictive strategies (Table 2).

Distribution of stage differed between strategies for colorectal cancer (Table 2). Assuming equivalence between values of zero and missing for N and M, as in the non-restrictive strategy, decreased dramatically the overall missingness of TNM stage grouping for colorectal cancer and affected the overall stage distribution. For instance, 25 431 (27.4% of data with observed stage) tumours were classified as stage III using the restrictive strategy and increased to 41 537 (30.2% of data with observed stage) using the non-restrictive strategy, mainly owing to the assumption of equivalence between missing and zero value of M in the first part of the algorithm. This difference was less pronounced for lung cancer, because of better completeness of the individual M component (Table 1). Lung cancer stage distribution was comparable between strategies (Table 2).

Using summary stage, variables in the non-restrictive strategy did not considerably improve the completeness of stage for either cancer site, indicating that most cases had fairly complete T, N, and M information before reaching this step or had all stage variables missing.

Age-standardised 1-year net survival for colorectal cancer was significantly lower using the non-restrictive strategy for all stages, particularly for the missing stage category. Differences in lung cancer survival between the two strategies were negligible (Table 3; Supplementary Appendix Figure S9). These figures reflect the differences between both strategies and how incomplete the individual stage components are.

## Discussion

This paper describes an algorithm to derive stage from multiple data sources. Recording of stage is now one of the Clinical Commissioning Group Outcome Indicators in England. However, it is rarely reported how this information is collected and then integrated into stage categories. We aim to adopt a standard approach to derive stage from multiple sources using a series of hierarchical rules. We have adapted it to specific cancer sites to illustrate its generalisability and highlight some data and cancer-specific issues.

In our example, the use of TNM Fifth Edition for colorectal cancer is justified to facilitate comparability of temporal trends (Royal College of Pathologists, 2014). There is a perceived increase in interobserver variability when assigning lymph node status using the shape criterion of the Seventh TNM Edition, rather than the size criterion of the Fifth Edition (Doyle and Bateman, 2012; Royal College of Pathologists, 2014). In England, the RPC recognises that some multidisciplinary teams – from which Clinical Audit stage data may be collected – use the Seventh Edition of TNM to stage colorectal cancers and that it might be requested in particular cases, such as those enrolled in clinical trials. There was poor individual information of the TNM edition used for staging colorectal cancer in the data sets we used. Given that there were some codes that are valid in TNM Seventh but not in TNM Fifth, we remain uncertain that all cases were staged using the Fifth Edition of TNM. There is conflicting evidence on the effect of using different editions of the TNM classification on the final staging (Nagtegaal *et al*, 2011; Doyle and Bateman, 2012). Nonetheless, comparing categories using different TNM editions may lead to stage migration, complicating comparisons of stage-specific outcomes. In contrast, the lung cancer data sets, in particular the Clinical Audit data, consistently reported an individual indicator of the edition of TNM used.

Distribution of colorectal cancer stage and stage-specific survival differed between strategies to define summary stage. Survival was lower for all stage categories using the non-restrictive strategy. Imputing all cases with missing M and/or N to a value of zero, as in the non-restrictive strategy, relies on very strong assumptions and may lead to misclassification, biased stage-specific survival estimates, and overly narrow variances. The missing stage categories contain a mixture of various stages, even though on average their prognosis is poorer than observed stage. The real stage distribution within the missing categories is different between strategies, as is their survival. The survival discrepancy between strategies was negligible for lung cancer. This is because there was more complete information on individual M component for lung (77.2%) than for colorectal cancer (58.8%). The restrictive approach is more conservative and keeps open, when necessary, the possibility of using specific approaches to deal with missing data, such as multiple imputation (White *et al*, 2011).

A particular limitation arises when applying the algorithm for staging tumours receiving neoadjuvant therapy. Pathological stage components are collected after neoadjuvant treatment, thus downgrading may occur. This issue may be addressed by making specific rules to deal with such tumours. This was not possible in our data given that information on neoadjuvant therapy is missing in the vast majority of cases from all available sources. Differences in aggressiveness of diagnostic investigation may also affect the comparability of stage-specific outcomes (Allemani *et al*, 2013).

We acknowledge potential limitations and have discussed the data issues and our assumptions. We encountered several issues in relation to coding of stage variables, inconsistencies in use of editions of TNM classification, conflicting stage information for single tumours, and a high proportion of missing data. We believe these may arise in other settings and data sets and have tried to address them in a transparent way, useful for other users of cancer staging information.

We have applied the algorithm to two cancer sites in a single country but aim for the hierarchical rules to be adaptable for other cancer sites and data sources in different countries, as the issue of inconsistently defined and reported stage categories is widespread in the current population-based cancer research (Ciccolallo *et al*, 2005; Walters *et al*, 2013a). The outcome will depend heavily on the quality of the specific data source but the general approach of prioritising information of highest quality, reporting sources of individual TNM variables, and reporting of assumptions when dealing with missing or inconsistent data is relevant to any cancer research using stage information. Descriptive results such as reported in Tables 1 and 2 are helpful in understanding the origins of summary stage and the reasons for shifts in stage distributions.

Validity of the information contained in cancer records remains a general issue. We believe it should be mandatory to have a relevant clinician at the health-care provider level ensuring that data collected are complete and truly reflect the information clinical decisions are based on. For each cancer case, it should be clear what classification was used to assign stage variables. As skilled clinicians are needed to collect and use stage information to make adequate medical decisions, there is also the need of people with standardised skills for recording and compiling of clinical information from medical records. The National Health System should make an effort to train and support such a workforce. Complete and accurate stage information is essential to assess cancer control policy and to understand inequalities in cancer management and cancer survival, at both national and international levels. We encourage cancer registries and health-care providers to clearly document the process for deriving stage grouping and reporting any data quality checks to validate this information. This information should be readily available for researchers.

## Figures and Tables

**Table 1:** Sources of valid T, N, and M components: completeness of variables and contribution to final staging

	**Data sets**		**Colorectal**		**Lung**	
**Variables**	**Audit**	**CAS**	**Completeness count (%)**	**Contribution count (%)**	**Completeness count (%)**	**Contribution count (%)**
**T component**						
pT	•		32 648 (19.9)	32 017 (19.5)^a^	15 427 (9.2)	15 427 (9.2)
Serosal involvement or perforation	•		5209 (3.2)	710 (0.4)	NA	NA
pT		•	77 997 (47.6)	52 761 (32.2)	14 933 (8.9)	5970 (3.6)
cT	•		48 204 (29.4)	16 113 (9.8)	107 171 (63.7)	89 265 (53.1)
Result from MRI	•		22 056 (13.5)	2295 (1.4)	NA	NA
cT		•	5773 (3.5)	754 (0.5)	7952 (4.7)	1217 (0.7)
iT		•	104 047 (63.5)	11 358 (6.9)	91 917 (54.7)	11 437 (6.8)
Missing T component				47 907 (29.2)		44 842 (26.7)
**N component**						
pN	•		32 666 (19.9)	32 518 (19.8)^a^	15 177 (9.0)	15 177 (9.0)
Count of positive lymph nodes	•		14 523 (8.9)	310 (0.2)	NA	NA
pN		•	74 212 (45.3)	49 351 (30.1)	15 180 (9.0)	6914 (4.1)
Count of positive lymph nodes		•	35 695 (21.8)	4656 (2.8)	3511 (2.1)	841 (0.5)
cN	•		50 108 (30.6)	16 141 (9.9)	107 300 (63.8)	88 336 (52.5)
Result from MRI	•		20 387 (12.4)	1319 (0.8)	NA	NA
cN		•	6576 (4.0)	1261 (0.8)	8085 (4.8)	1274 (0.8)
iN		•	101 453 (61.9)	9574 (5.8)	91 693 (54.5)	11 349 (6.7)
Missing N component				48 785 (29.8)		44 267 (26.3)
**M component**						
cM	•		51 542 (31.4)	50 548 (30.8)^a^	107 057 (63.7)	107 057 (63.7)
Distant metastasis	•		17 890 (10.9)	8727 (5.3)	NA	NA
Result from liver CT	•		75 714 (46.2)	1064 (0.6)	NA	NA
cM		•	13 212 (8.1)	6548 (4.0)	8987 (5.3)	1894 (1.1)
pM	•		10 333 (6.3)	3248 (2.0)	10 845 (6.4)	648 (0.4)
pM		•	10 936 (6.7)	4170 (2.5)	9675 (5.8)	2944 (1.8)
iM		•	67 948 (41.5)	18 800 (11.5)	92 930 (55.3)	14 197 (8.4)
Clinical examination				3294 (2.0)		3056 (1.8)
Missing M component				67 516 (41.2)		38 362 (22.8)
**Summary stage**^b^						
pStage	•		30 239 (18.4)^c^	2206 (1.3)^c^	16 946 (10.1)	20 (<0.01)
cStage	•		80 036 (48.8)^c^	1098 (0.7)^c^	105 410 (62.7)	485 (0.3)
pStage		•	4351 (2.7)	38 (<0.01)	2117 (1.3)	41 (<0.01)
cStage		•	508 (0.3)	5 (<0.01)	679 (0.4)	7 (<0.01)
iStage		•	57 596 (35.1)	1189 (0.7)	85 267 (50.7)	1520 (0.9)
Stage		•	108 105 (66.0)^d^	4003 (2.4)^d^	NA	NA
Total				163 915		168 158

Abbreviations: CAS=Cancer Analysis System; M=metastases; MRI=magnetic resonance imaging; N=lymph nodes; NA=not available; T=tumour. Colorectal and lung cancer diagnoses in England, 2008–2012. Prefixes: p: pathological; c: clinical; i: integrated (origin may be pathological, clinical, highest value, or simply flagged by the registry).

a Some zero values for this variable are replaced by positive values in the next step of the algorithm, where the contribution to final staging is made. Therefore, completeness of this variable does not equal its contribution to final staging.

b Summary stage variables contribute to non-restrictive strategy only.

c Dukes stage from Audit.

d Dukes stage from CAS.

**Table 2:** Overall stage grouping by cancer, year of diagnosis, and staging strategy

	**Year of diagnosis**					
	2008 count (%*)	2009 count (%*)	2010 count (%*)	2011 count (%*)	2012 count (%*)	Total count (%*)
**Colorectal cancer**						
**Non-restrictive strategy**						
Missing stage	6996 (22.1)	6408 (19.8)	5114 (15.7)	4777 (14.3)	3191 (9.4)	26 486 (16.2)
Observed stage	24 630 (77.9)	25 939 (80.2)	27 518 (84.3)	28 708 (85.7)	30 634 (90.6)	137 429 (83.8)
I	3774 (15.3)	4159 (16.0)	4602 (16.7)	5043 (17.6)	5803 (18.9)	23 381 (17.0)
II	7631 (31.0)	7612 (29.3)	7764 (28.2)	7944 (27.7)	8195 (26.8)	39 146 (28.5)
III	7890 (32.0)	8212 (31.7)	8495 (30.9)	8508 (29.6)	8432 (27.5)	41 537 (30.2)
IV	5335 (21.7)	5956 (23.0)	6657 (24.2)	7213 (25.1)	8204 (26.8)	33 365 (24.3)
**Restrictive strategy**						
Missing stage	20 948 (66.2)	19 360 (59.9)	15 071 (46.2)	10 749 (32.1)	5009 (14.8)	71 137 (43.4)
Observed stage	10 678 (33.8)	12 987 (40.1)	17 561 (53.8)	22 736 (67.9)	28 816 (85.2)	92 778 (56.6)
I	1046 (9.8)	1434 (11.0)	2333 (13.3)	3637 (16.0)	5117 (17.8)	13 567 (14.6)
II	2175 (20.4)	2684 (20.7)	4169 (23.7)	5883 (25.9)	7645 (26.5)	22 556 (24.3)
III	2759 (25.8)	3455 (26.6)	4839 (27.6)	6408 (28.2)	7970 (27.7)	25 431 (27.4)
IV	4698 (44.0)	5414 (41.7)	6220 (35.4)	6808 (29.9)	8084 (28.1)	31 224 (33.7)
Total	31 626	32 347	32 632	33 485	33 825	163 915
**Lung cancer**						
**Non-restrictive strategy**						
Missing stage	11 498 (35.9)	8242 (25.0)	5622 (16.8)	3938 (11.4)	2441 (6.9)	31 741 (18.9)
Observed stage	20 509 (64.1)	24 765 (75.0)	27 846 (83.2)	30 463 (88.6)	32 834 (93.1)	136 417 (81.1)
I	2888 (14.1)	3560 (14.4)	3713 (13.3)	4092 (13.4)	4871 (14.8)	19 124 (14.0)
II	1303 (6.4)	1661 (6.7)	2221 (8.0)	2509 (8.2)	2764 (8.4)	10 458 (7.7)
III	6338 (30.9)	7204 (29.1)	7030 (25.2)	7211 (23.7)	7623 (23.2)	35 406 (26.0)
IV	9980 (48.7)	12 340 (49.8)	14 882 (53.4)	16 651 (54.7)	17 576 (53.5)	71 429 (52.4)
**Restrictive strategy**						
Missing stage	13 661 (42.7)	10 143 (30.7)	7321 (21.9)	5121 (14.9)	3046 (8.6)	39 292 (23.4)
Observed stage	18 346 (57.3)	22 864 (69.3)	26 147 (78.1)	29 280 (85.1)	32 229 (91.4)	128 866 (76.6)
I	2223 (12.1)	2952 (12.9)	3237 (12.4)	3781 (12.9)	4703 (14.6)	16 896 (13.1)
II	1060 (5.8)	1455 (6.4)	2030 (7.8)	2383 (8.1)	2696 (8.4)	9624 (7.5)
III	5357 (29.2)	6434 (28.1)	6531 (25.0)	6894 (23.5)	7437 (23.1)	32 653 (25.3)
IV	9706 (52.9)	12 023 (52.6)	14 349 (54.9)	16 222 (55.4)	17 393 (54.0)	69 693 (54.1)
Total	32 007	33 007	33 468	34 401	35 275	168 158

Note: %*: Percentages for stages I to IV represent the proportion of observed stage data, excluding observations with missing stage. Colorectal and lung cancer diagnoses in England, 2008–2012.

**Table 3:** Age-standardised estimates of 1- and 5-year net survival by cancer, stage, and staging strategy

	**Non-restrictive staging strategy**	**Restrictive staging strategy**
**Stage**	**NS (CI)**	**NS (CI)**
**Colorectal cancer**		
**One-year net survival**		
I	0.979 (0.976, 0.981)	0.982 (0.978, 0.985)
II	0.936 (0.933, 0.939)	0.949 (0.945, 0.952)
III	0.880 (0.877, 0.883)	0.898 (0.893, 0.902)
IV	0.495 (0.489, 0.501)	0.510 (0.504, 0.515)
Missing	0.605 (0.599, 0.612)	0.777 (0.774, 0.780)
**Five-year net survival**		
I	0.952 (0.944, 0.960)	0.957 (0.945, 0.969)
II	0.849 (0.843, 0.855)	0.861 (0.852, 0.871)
III	0.638 (0.632, 0.645)	0.665 (0.655, 0.674)
IV	0.152 (0.146, 0.157)	0.158 (0.152, 0.164)
Missing	0.414 (0.406, 0.423)	0.619 (0.614, 0.624)
**Lung cancer**		
**One-year net survival**		
I	0.843 (0.837, 0.848)	0.852 (0.846, 0.858)
II	0.685 (0.675, 0.695)	0.693 (0.683, 0.704)
III	0.431 (0.425, 0.437)	0.439 (0.433, 0.445)
IV	0.182 (0.179, 0.185)	0.183 (0.180, 0.186)
Missing	0.256 (0.250, 0.262)	0.298 (0.293, 0.303)
**Five-year net survival**		
I	0.542 (0.531, 0.554)	0.541 (0.529, 0.554)
II	0.325 (0.310, 0.340)	0.325 (0.309, 0.341)
III	0.099 (0.094, 0.104)	0.100 (0.095, 0.105)
IV	0.025 (0.024, 0.027)	0.026 (0.024, 0.028)
Missing	0.093 (0.088, 0.098)	0.125 (0.120, 0.130)

Abbreviations: CI, confidence interval; NS, net survival. Diagnoses in England, 2008–2012.
