# Supplementary material for: Deriving stage at diagnosis from multiple population-based sources: colorectal and lung cancer in England
Source: Br J Cancer. 2016 Jun 21;115(3):391–400. doi: 10.1038/bjc.2016.177 (PMC4973150; doi:10.1038/bjc.2016.177)
Supplement: Supplementary Appendix [file bjc2016177x1.pdf]

Supplementary Web-Appendix

Contents

Web-Appendix Figure 1. Deriving T component of TNM stage for colorectal cancer, England, 2008-2012....................... 2

Web-Appendix Figure 2. Deriving N component of TNM stage for colorectal cancer, England, 2008-2012. 3

Web-Appendix Figure 3. Deriving M component of TNM stage for colorectal cancer, England, 2008-2012. 4

Web-Appendix Figure 4. Deriving TNM stage for lung cancer using the restrictive strategy, England, 2008-2012. 5

Web-Appendix Figure 5. Deriving TNM stage for lung cancer using the non-restrictive strategy, England, 2008-2012.... 6

Web-Appendix Figure 6. Deriving T component of TNM stage for lung cancer, England, 2008-2012............................... 7

Web-Appendix Figure 7. Deriving N component of TNM stage for lung cancer, England, 2008-2012. 8

Web-Appendix Figure 8. Deriving M component of TNM stage for lung cancer, England, 2008-2012. 9

Web-Appendix Figure 9. Age standardised net survival by TNM stage, England 2008-2012 10

Notes:.. 10

Web-Appendix Figure 1. Deriving T component of TNM stage for colorectal cancer, England, 2008-2012.

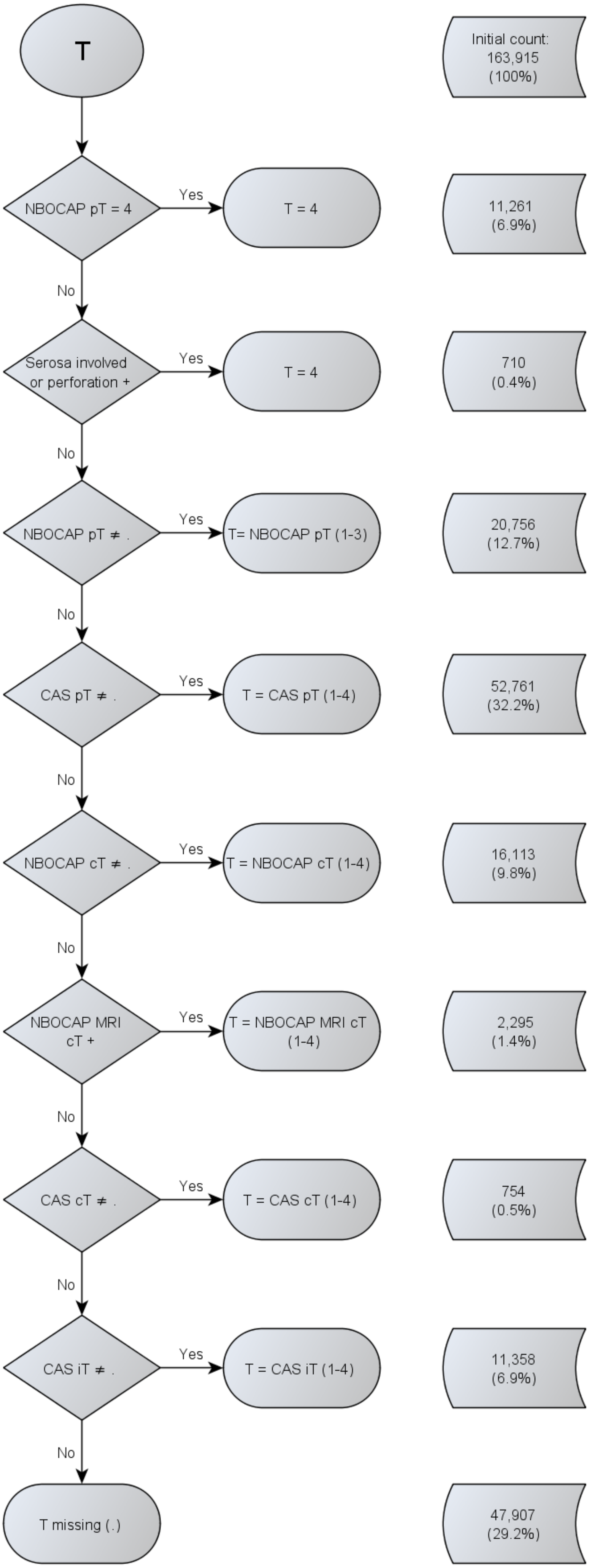

Web-Appendix Figure 2. Deriving N component of TNM stage for colorectal cancer, England, 2008-2012.

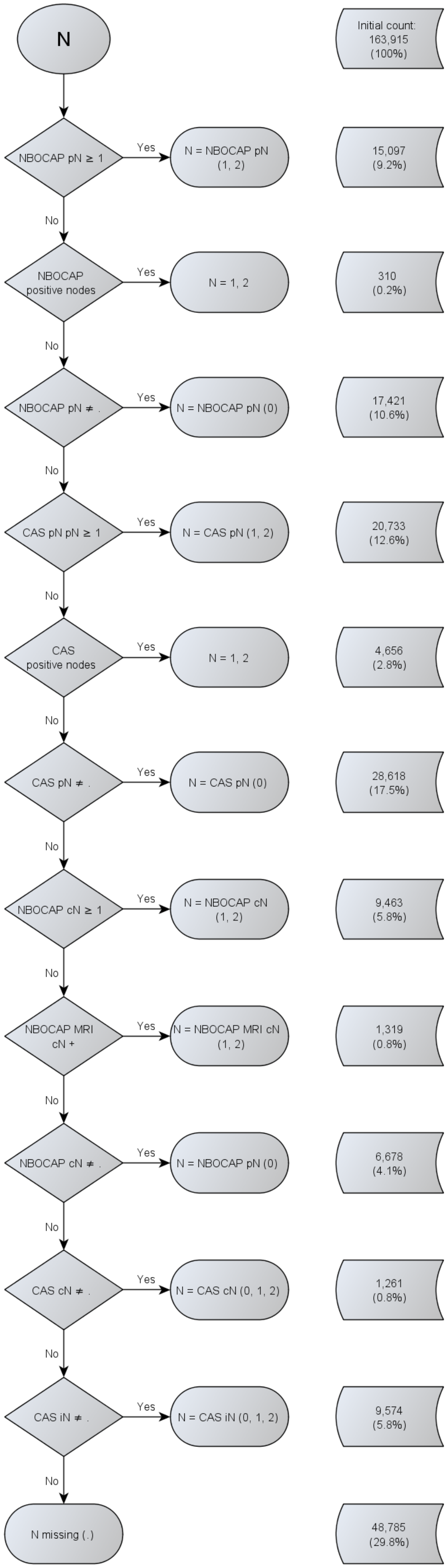

Web-Appendix Figure 3. Deriving M component of TNM stage for colorectal cancer, England, 2008-2012.

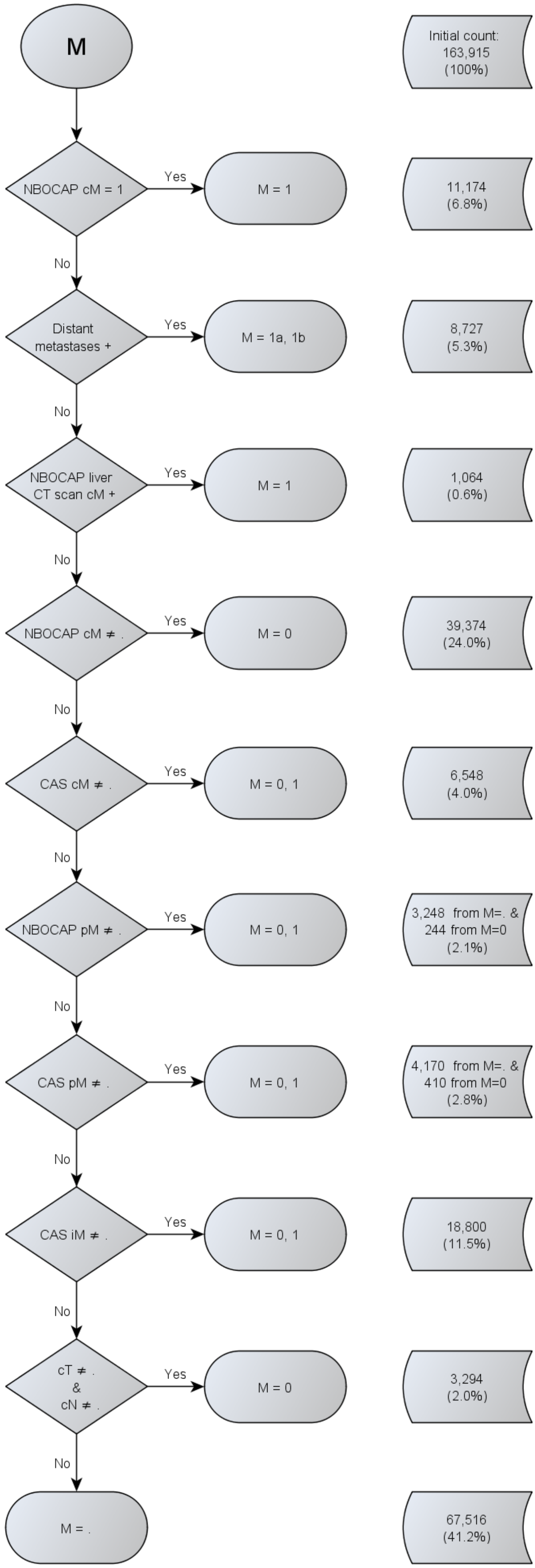

Web-Appendix Figure 4. Deriving TNM stage for lung cancer using the restrictive strategy, England, 2008-2012.

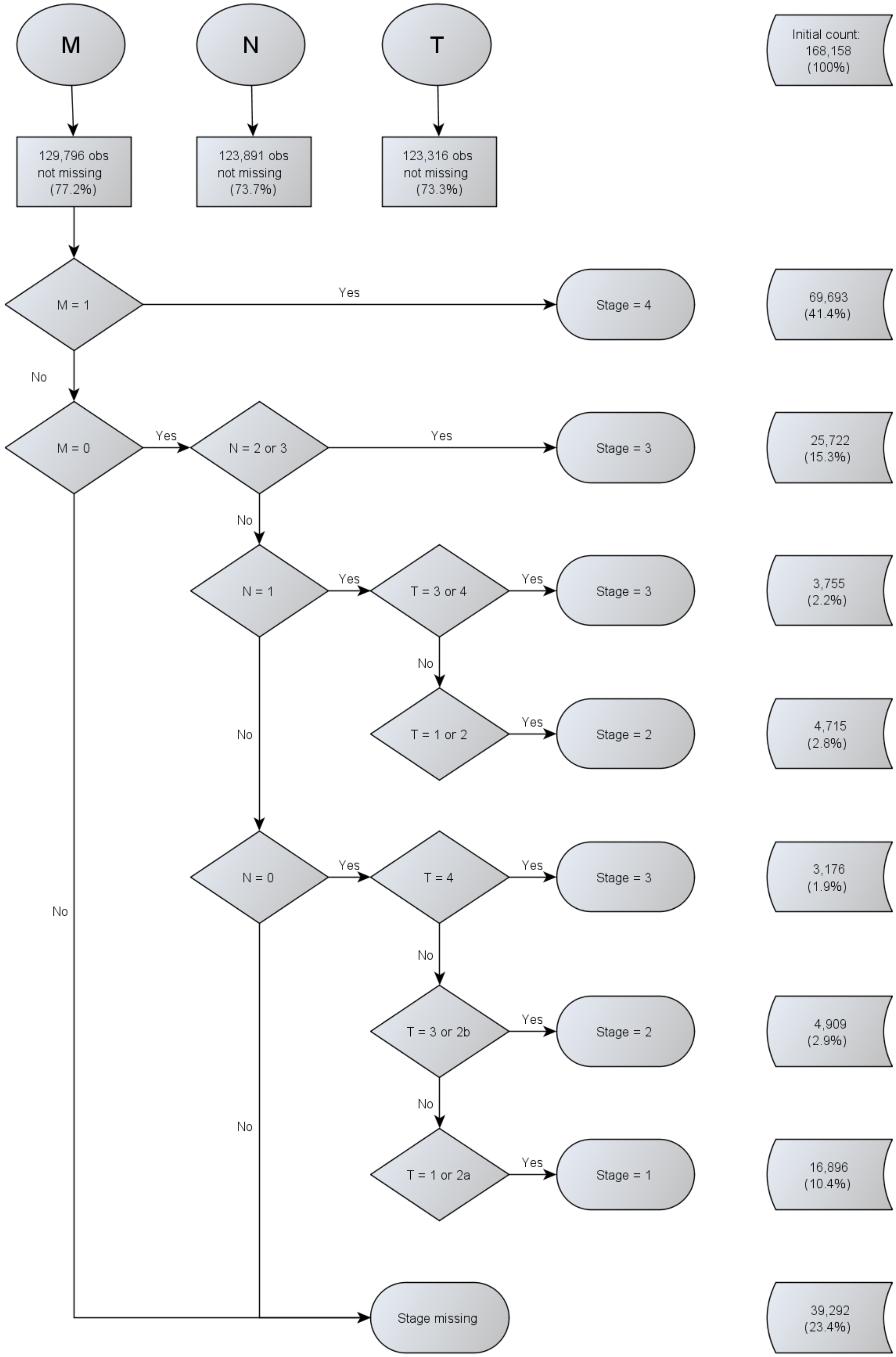

Web-Appendix Figure 5. Deriving TNM stage for lung cancer using the non-restrictive strategy, England, 2008-2012.

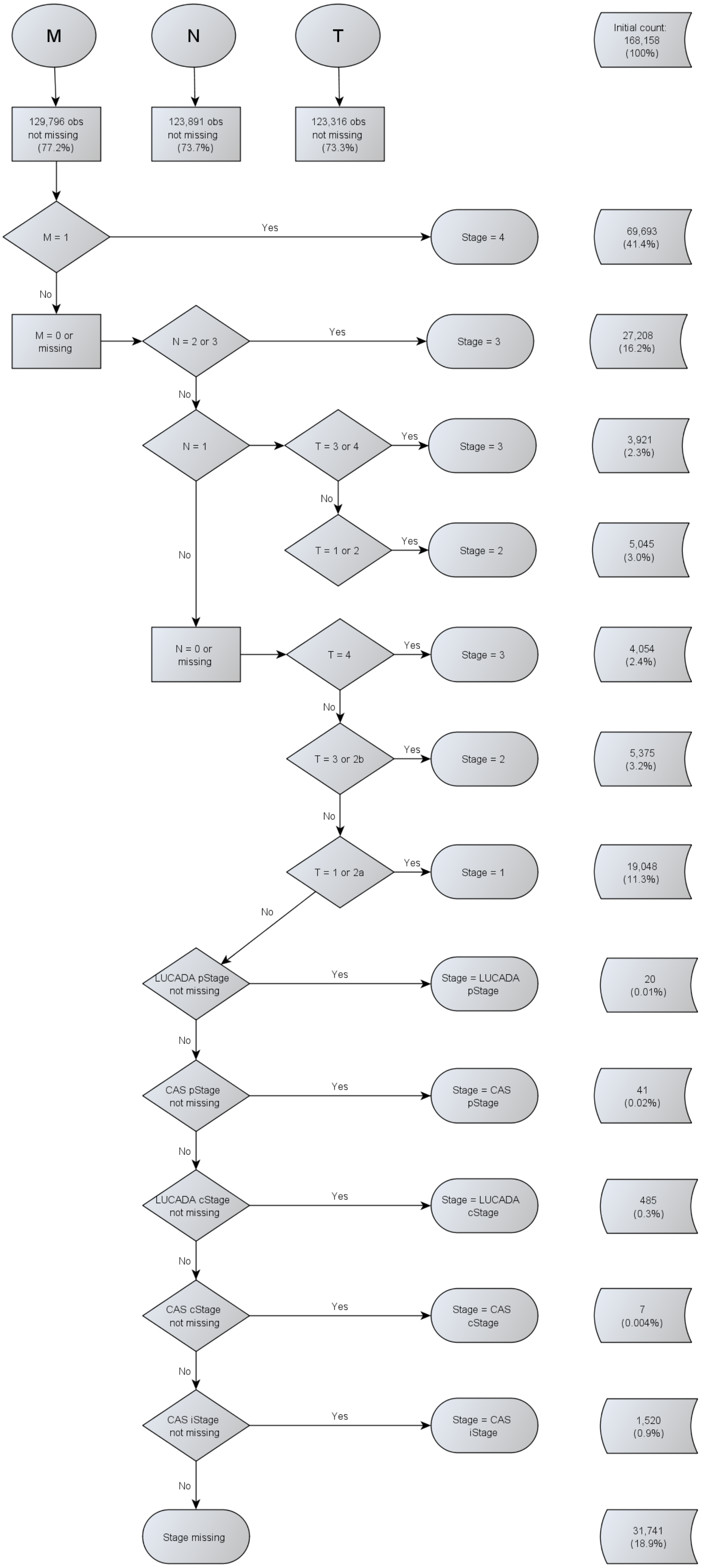

Web-Appendix Figure 6. Deriving T component of TNM stage for lung cancer, England, 2008-2012.

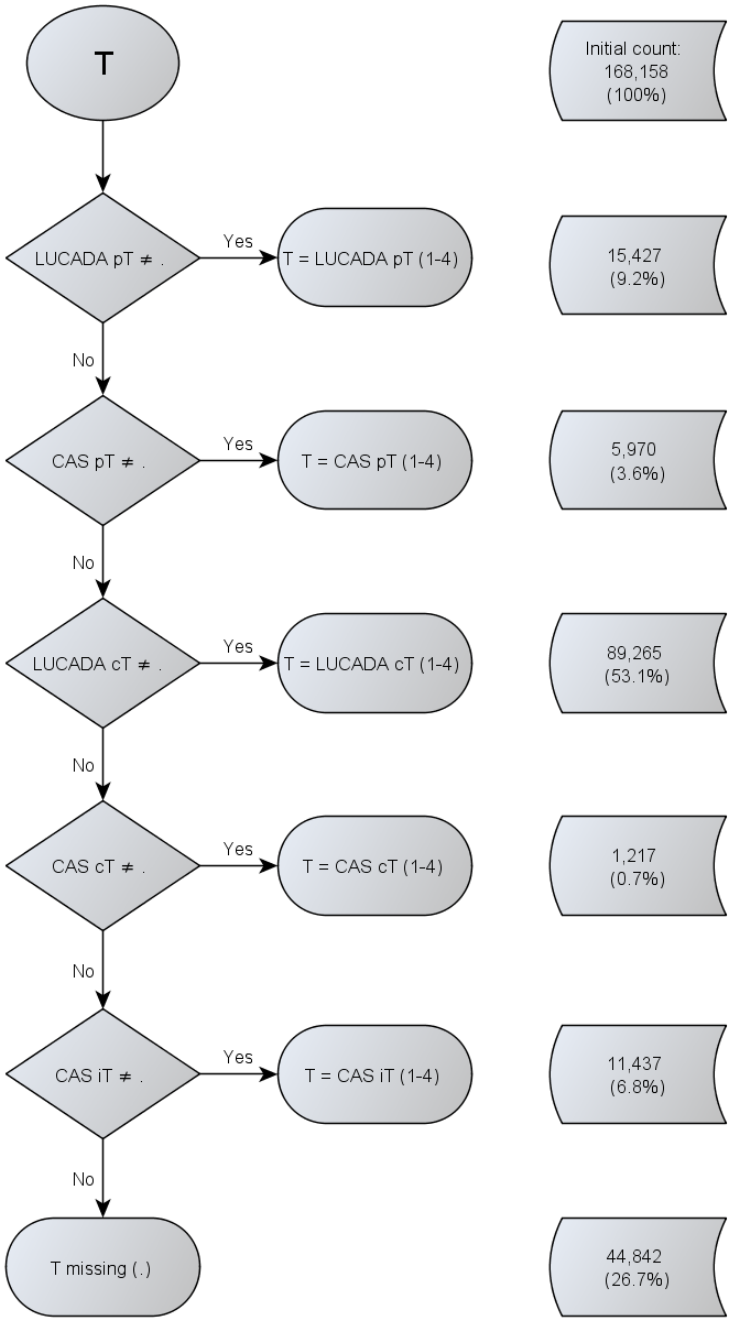

Web-Appendix Figure 7. Deriving N component of TNM stage for lung cancer, England, 2008-2012.

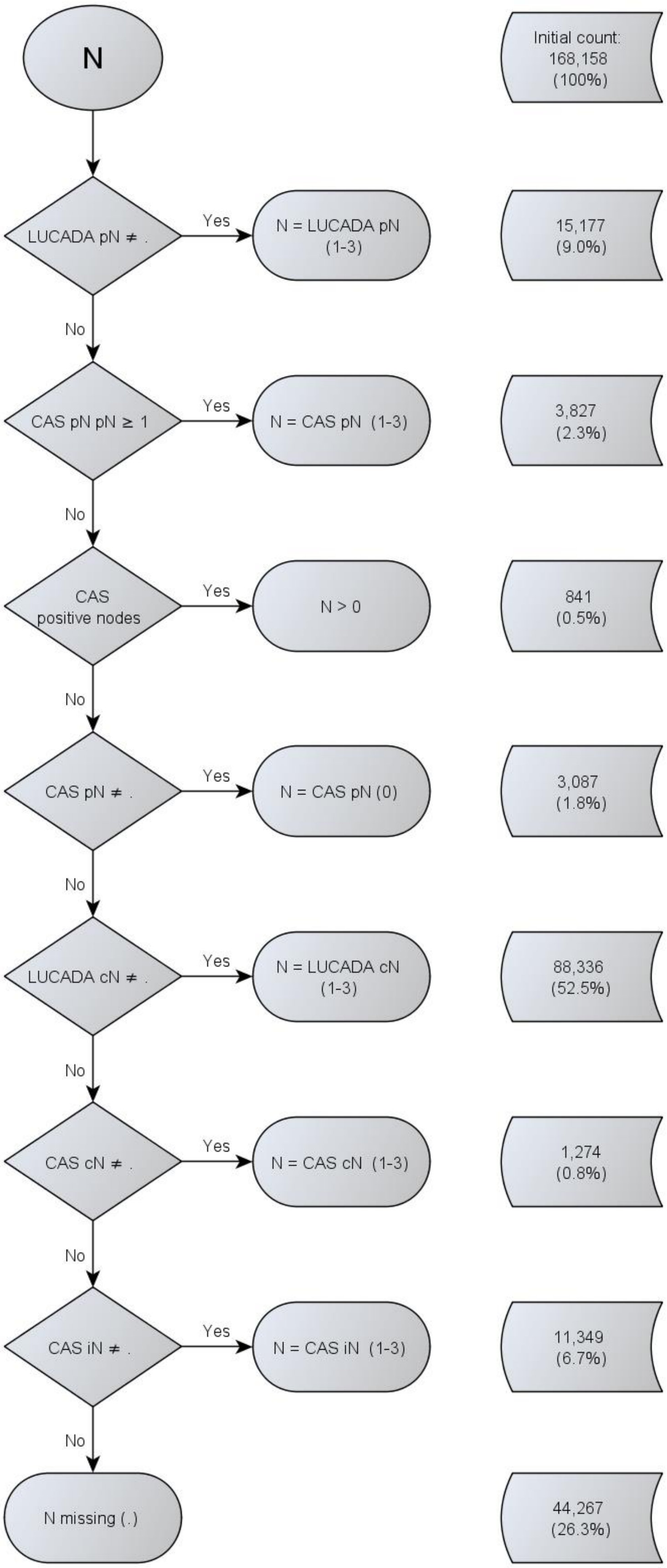

Web-Appendix Figure 8. Deriving M component of TNM stage for lung cancer, England, 2008-2012.

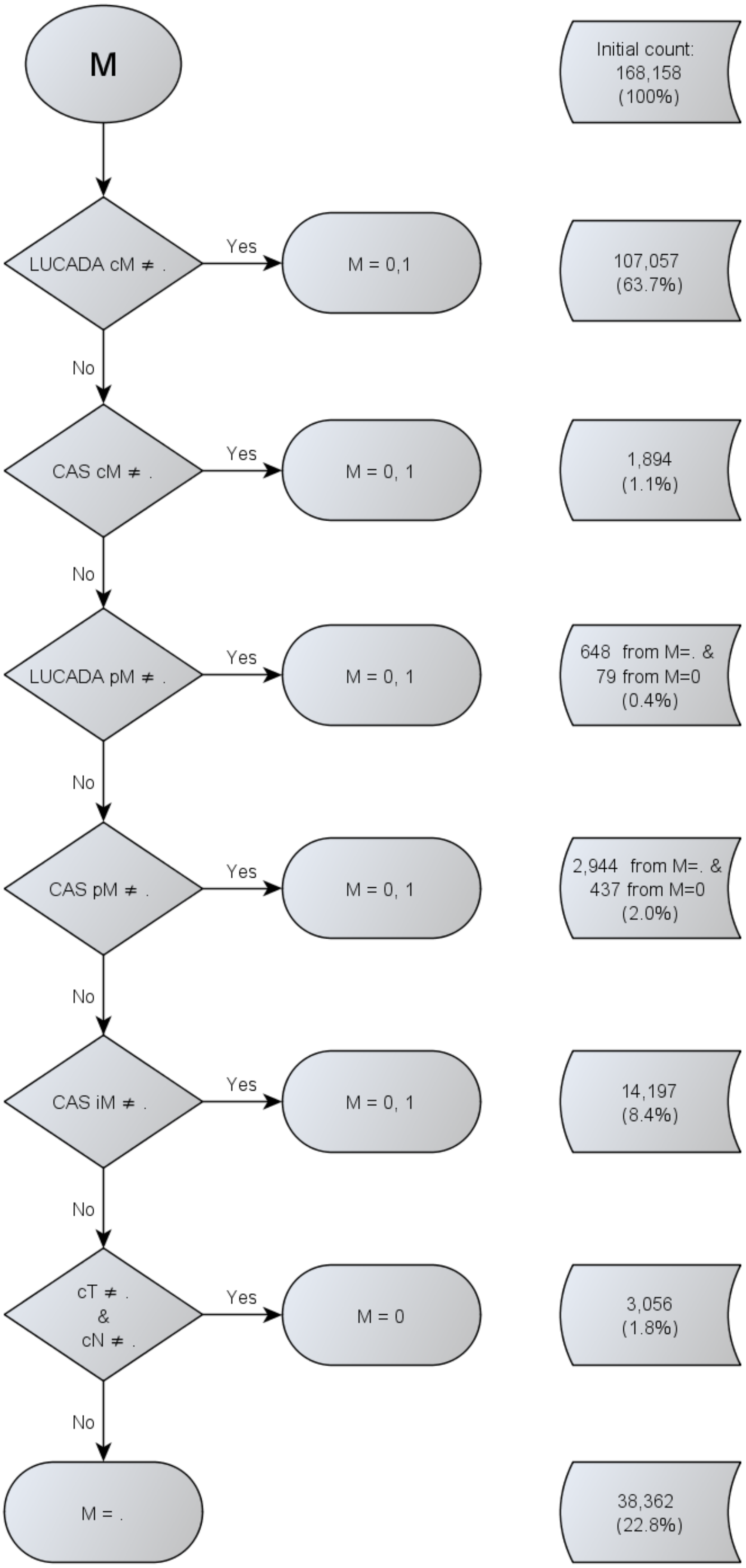

Web-Appendix Figure 9. Age standardised net survival by TNM stage, England 2008-2012

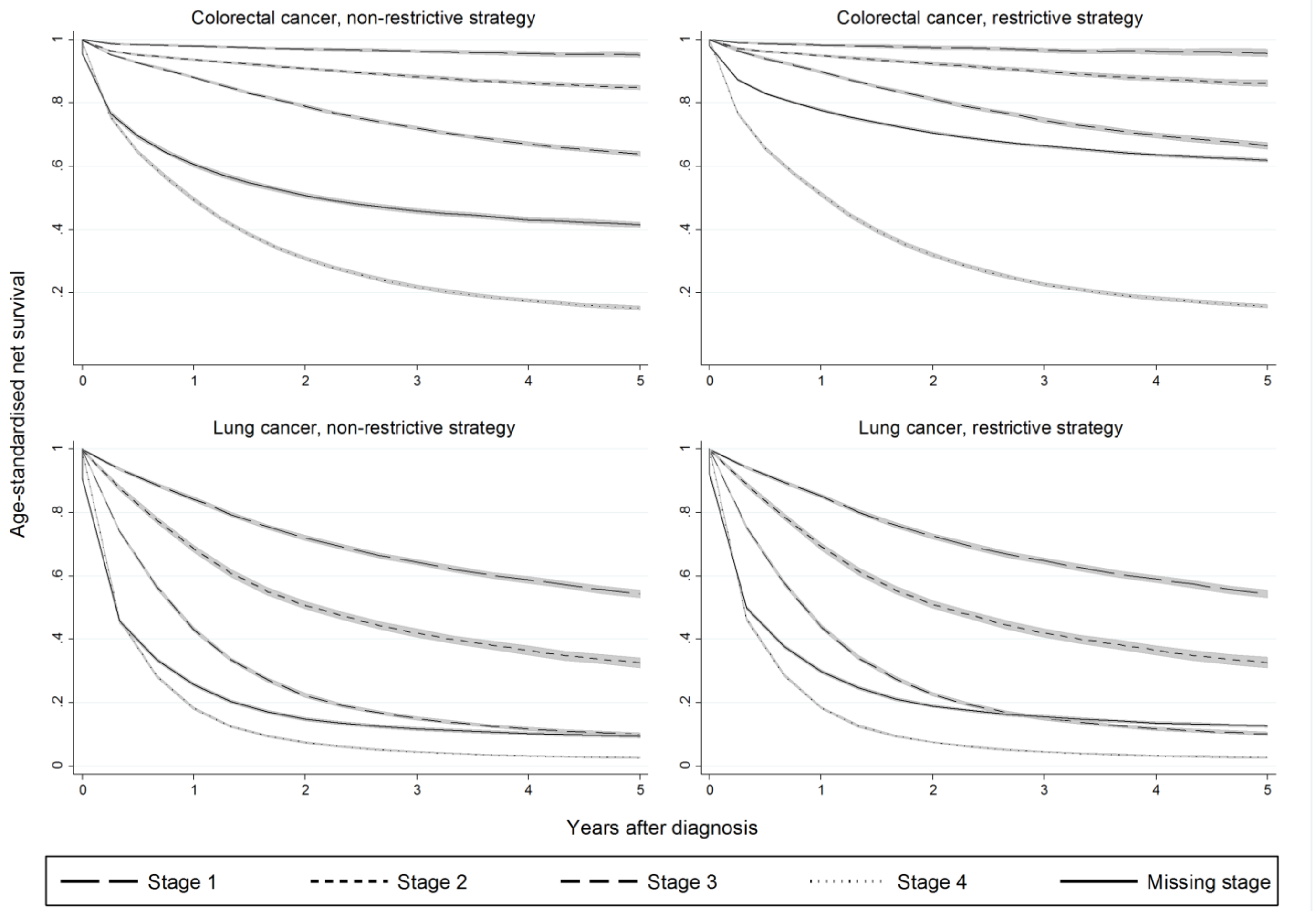

Notes:

T: Tumour component of TNM stage
NBOCAP: National Bowel Cancer Project
pT: Pathological T
%: Percent
+: Positive
≠: Not equal to
.: Missing
CAS: Cancer Analysis System
cT: Clinical T
MRI: Magnetic Resonance Imaging
iT: Integrated T
N: Lymph nodes component of TNM stage
pN: Pathological N
≥: Greater than or equal to
cN: Clinical N
iN: Integrated N
M: Distant Metastases component of TNM stage
cM: Clinical M
CT: Computerised Tomography scan
pM: Pathological M
iM: Integrated M
pStage: Pathological TNM Stage
cStage: Clinical TNM Stage
iStage: Integrated TNM Stage
LUCADA: Lung Cancer Audit Database
